# Advancements in the synergy of isothermal amplification and CRISPR-cas technologies for pathogen detection

**DOI:** 10.3389/fbioe.2023.1273988

**Published:** 2023-10-10

**Authors:** Xiaolei Mao, Minghui Xu, Shuyin Luo, Yi Yang, Jiaye Zhong, Jiawei Zhou, Huayan Fan, Xiaoping Li, Zhi Chen

**Affiliations:** ^1^ Key Laboratory of Pollution Exposure and Health Intervention of Zhejiang Province, Shulan International Medical College, Zhejiang Shuren University, Hangzhou, China; ^2^ Faculty of Medicine, Macau University of Science and Technology, Macau, China; ^3^ Shulan International Medical College, Zhejiang Shuren University, Hangzhou, China

**Keywords:** recombinase polymerase amplification, recombinase-aid amplification, CRISPR-cas, Cas12, Cas13, rapid inspection technology improvement, sherlock, DETECTR

## Abstract

In the realm of pathogen detection, isothermal amplification technology has emerged as a swift, precise, and sensitive alternative to conventional PCR. This paper explores the fundamental principles of recombinase polymerase amplification (RPA) and recombinase-aid amplification (RAA) and reviews the current status of integrating the CRISPR-Cas system with RPA/RAA techniques. Furthermore, this paper explores the confluence of isothermal amplification and CRISPR-Cas technology, providing a comprehensive review and enhancements of existing combined methodologies such as SHERLOCK and DETECTR. We investigate the practical applications of RPA/RAA in conjunction with CRISPR-Cas for pathogen detection, highlighting how this integrated approach significantly advances both research and clinical implementation in the field. This paper aims to provide readers with a concise understanding of the fusion of RPA/RAA and CRISPR-Cas technology, offering insights into their clinical utility, ongoing enhancements, and the promising prospects of this integrated approach in pathogen detection.

## 1 Introduction

Pathogens, encompassing viruses, bacteria, fungi, and other microorganisms, pose a constant threat by invading the human body, causing infections, and propagating diseases. Timely and precise pathogen detection constitutes a pivotal facet of clinical diagnostics and biotechnological applications. The Polymerase Chain Reaction (PCR) has long stood as the gold standard for nucleic acid amplification in scientific research and diagnostics, comprising denaturation, annealing, and extension steps, albeit requiring specialized and bulky equipment (Fan et al., 2020). This limitation has restricted its accessibility in resource-constrained regions (Srivastava and Prasad, 2023). In recent years, isothermal amplification technology (IAT) has emerged as an alternative for detecting various pathogens, offering the advantage of simplicity and versatility, enabling its use in both field and laboratory settings (Boonbanjong et al., 2022). Notably, IAT excels over PCR in terms of sensitivity and specificity (Lu et al., 2020; Yu et al., 2020). Prominent techniques in IAT encompass loop-mediated isothermal amplification (LAMP), RPA, and RAA (Yan et al., 2014).

Concurrently, Clustered Regularly Interspaced Short Palindromic Repeats (CRISPR)-based diagnostics have gained prominence as the next-generation molecular diagnostic approach. Among CRISPR-associated proteins, Cas12 and Cas13 proteins have been extensively harnessed for DNA and RNA detection (Guk et al., 2022).

Cas13a, belonging to the Type VI effector protein family within the CRISPR system, has garnered particular attention and has found application in molecular diagnostics, gene therapy, and pathogen detection, spanning viruses, bacteria, parasites, *chlamydia*, and fungi (Knott et al., 2017). The CRISPR-Cas13a system has successfully detected respiratory, DNA, and hemorrhagic fever viruses, demonstrating its potential as a next-generation diagnostic tool. Although CRISPR technology can independently target specific nucleic acid sequences, its accuracy falls short (Gootenberg et al., 2017). Hence, the amalgamation of isothermal recombinase amplification with CRISPR-Cas technology holds substantial promise.

In the realm of combined technology, Specific High Sensitivity Reporter Gene Unlock (SHERLOCK) and DNA Endonuclease-Targeted CRISPR Trans Reporter (DETECTR) have emerged as mature methodologies. These approaches offer multiplicity, portability, and cost-effective sensitivity in detection. However, their combined application presents challenges, primarily due to the need for a two-step operation, potentially leading to contamination after the isothermal amplification process. The synergy of isothermal amplification and CRISPR technology for sensitive and specific nucleic acid detection remains a formidable challenge due to compatibility issues (Uno et al., 2023). Nonetheless, it represents a significant advancement in the field of pathogen detection, holding the potential to revolutionize the landscape of pathogen diagnostics.

## 2 Isothermal amplification techniques

The foremost isothermal amplification technique in use is LAMP, which has seen successful applications in numerous assays. However, LAMP presents certain challenges, notably its reliance on higher temperatures and the intricacy associated with primer design. Consequently, there has been a shift towards employing RPA and RAA techniques for pathogen detection. These emerging technologies hold substantial promise across diverse domains, encompassing viruses, bacteria, and parasites responsible for human and animal diseases, as well as applications in food safety and the detection of plant diseases.

Continuous advancements and research efforts aimed at developing thermostable nucleic acid amplification reagents for RAA and RPA have paved the way for innovative applications, notably Reverse Transcriptional Polymerase Amplification (RT-RPA) and Reverse Transcriptional Recombinase-Aided Amplification (RT-RAA). These methodologies have gained traction for studying RNA viruses (Wang H. et al., 2022; Cui et al., 2022).

### 2.1 Recombinase polymerase amplification

RPA relies on the coordinated action of two essential enzymes: the recombinase T4 bacteriophage enzyme and *Bacillus subtilis* Pol I (Piepenburg et al., 2006). The RPA reaction system encompasses several critical components, including amplification templates, primers, and various raw materials (Tan et al., 2022). RPA stands out as an exquisitely sensitive and selective isothermal amplification technique, operating effectively within a temperature range of 37°C–42°C. It boasts minimal sample preparation requirements and the remarkable capability to amplify as few as 1 to 10 DNA target copies within a mere 20 min (Lobato and O'Sullivan, 2018).

The underlying principle of RPA hinges on the initiation of a complex series of events. Recombinase first binds to primers when ATP is present, forming protein-nucleic acid polymers that actively search for complementary target sequences. Upon locating the target DNA template, ATP hydrolysis provides the necessary energy, allowing the recombinase to synthesize a new DNA strand with the assistance of DNA polymerase. Simultaneously, the single-strand DNA-binding protein (SSB) binds to the single strand of the displaced DNA, effectively preventing the DNA template from re-forming a double strand. This RPA-ssDNA platform plays a pivotal role in protecting and repairing stalled or collapsed replication forks during replication stress by activating the master ATR kinase (Marechal and Zou, 2015).

Remarkably, RPA-based amplification permits reactions to proceed within rudimentary setups such as a water bath or portable heater, achieving the requisite temperature conditions (Cherkaoui et al., 2021). Building upon RPA, RT-RPA emerges as a valuable extension. RT-RPA initially employs reverse transcriptase to transcribe RNA into complementary DNA (cDNA). Subsequently, the recombinase collaborates with primer DNA to form a complex that infiltrates the RNA-cDNA double-stranded nucleic acid template. This action leads to the opening of the double-strand at the invasion site, with the single-chain binding protein preserving the template in the open-chain state. The recombinase/primer complex then scans for a double-stranded region, and upon finding a complementary sequence on the template that matches the primer, the complex disintegrates. Subsequently, DNA polymerase binds to the 3′end of the primer, initiating the synthesis of a new strand. This newly synthesized strand serves as a template, resulting in exponential growth of the final amplified product, ultimately achieving the expansion of the target gene.

### 2.2 Recombinase-aided amplification

RAA stands out as an innovative isothermal amplification technique, characterized by its rapid completion within 42 min at temperatures ranging from 30°C to 37°C. This approach hinges on the collaborative actions of three essential enzymes: SSB, recombinase, and DNA polymerases (Li et al., 2020; Tu et al., 2022). RAA represents a novel and expeditious nucleic acid amplification method that operates effectively at lower temperatures, typically 37°C. The reaction proceeds swiftly, yielding amplified products within just 30 min. Its burgeoning popularity for detecting various pathogenic microorganisms can be attributed to its straightforward primer design, rapid amplification kinetics, heightened sensitivity, minimal equipment requirements, user-friendly operation, visual result output, and other inherent advantages (Tu et al., 2021).

Notably, RAA has demonstrated successful applications in the detection of adenovirus (Wang RH. et al., 2019), Coxsackie virus, enterovirus, and respiratory syncytial virus, as attested by the available literature (Qi et al., 2019). Building on RAA, RT-RAA has emerged as a cutting-edge nucleic acid detection methodology. This approach involves the introduction of recombinase and single-stranded binding protein into the amplification system. RT-RAA achieves rapid amplification at 39°C, with results obtainable within minutes (Nie et al., 2022). A key advantage of RT-RAA is its capacity to directly utilize RNA as a template for pathogen detection. This feature liberates the amplified product from location constraints, enabling detection not only within laboratory settings, utilizing common laboratory instruments, but also in the field using portable devices (Wang Y. et al., 2022). The evaluation of RT-RAA encompasses a comprehensive assessment, including different primer compositions, specificity, and assay sensitivity (Piepenburg et al., 2006).

Presently, as RPA and RAA technologies continue to mature and undergo refinement through ongoing research efforts, they serve as foundational elements for the development of new molecular diagnostic technologies. These technologies also bolster fundamental research in molecular biology. The future holds promise for further advancements, including potential integration with other detection methods or the development of cost-effective materials. Compared to combining CRISPR with LAMP, RPA, and RAA, the former offers greater convenience and minimizes the occurrence of false positives (Zhang X. et al., 2022). It is anticipated that as RPA and RAA technologies are further explored and refined, they will assume even more prominent roles in the future of molecular diagnostics.

## 3 CRISPR and CRISPR cas systems

The CRISPR-Cas system has emerged as a focal point of biological research today. Initially observed in archaea in 1993, CRISPR sequences have since been identified in an expanding array of bacterial and archaeal genomes. Subsequently, in the early 21st century, scientists began uncovering the significance of CRISPR and its role within the immune system. Comparative genetic analysis revealed that CRISPR, in conjunction with Cas proteins, constitutes an acquired immune system, akin to the eukaryotic RNA interference (RNAi) system, aimed at safeguarding prokaryotic cells from external threats (Ishino et al., 2018). The CRISPR-Cas system has played a pivotal role in advancing the field of gene editing and has found widespread applications across the life sciences. As researchers continue to enhance its performance, they have devised a diverse range of tools capable of targeting a broad spectrum of genetic elements while maintaining exceptional precision (Liu G. et al., 2022).

Fundamentally, the CRISPR-Cas system serves as a prokaryotic adaptive immune system that identifies and cleaves foreign nucleic acids (Marraffini and Sontheimer, 2008). This system can be categorized into two main classes: Class 1 and Class 2. Class 1 employs multi-protein complexes to neutralize foreign nucleic acids, encompassing type I, type III, and type IV systems. In contrast, Class 2 primarily relies on a single protein to achieve its function, encompassing types II, V, and VI (Makarova et al., 2020). Table 1 provides a comprehensive overview of common CRISPR-Cas nucleases, including details such as nuclease types, protein sizes, domains, target preferences, and Protospacer Adjacent Motif/Protospacer Flanking Sequence (PAM/PFS) recognition motifs, offering a clearer understanding of the diversity within the CRISPR-Cas system. This table serves as a valuable reference, offering insights into the rich diversity and functionality encapsulated within the CRISPR-Cas system, further fueling its widespread applications across scientific disciplines.

**TABLE 1:** Classification of CRISPR-Cas proteins.

Cas	Type	Protein size	Structure domains	Target nucleic acids	PAM/PFS	Amplification methods	References
Cas9	Type II	1000–1600	HNH, RuvC	DNA	5ʹ-NGG or 5ʹ-NAG	CAS-EXPAR	Hsu et al. (2014), Paul and MontoyaCRISPR-Cas12a (2020), Yuan et al. (2022)
Cas12a	Type V	1300	Similar to RuvC	DNA	5′-TTTN (T-rich sequences at the 5′end)	RPA, PCR/RT-PCR	Paul and MontoyaCRISPR-Cas12a (2020), Yuan et al. (2022)
Cas12b	Type V	1100	RuvC	DNA	PAM not required for ssDNA, required for dsDNA	LAMP	Strecker et al. (2019), Yuan et al. (2022)
Cas13a	Type VI	1250	Two HEPN	RNA	3′non-G PFS preference (except Lwa and LbuCas13a)	RPA	Zhang and You (2020), Yuan et al. (2022), Zhao et al. (2022)
Cas13b	Type VI	1150	Two HEPN	RNA	Results exist at the 3′end of crRNA in comparison with the 5′end of Cas13a, Cas13c, and Cas13d	RPA	Sma et al. (2017), Kavuri et al. (2022), Yuan et al. (2022)
Cas13c	Type VI	1120	Two HEPN	RNA	No PFS preference	RPA	Kavuri et al. (2022), Yuan et al. (2022)
Cas13d	Type VI	930	Two HEPN	RNA	No PFS preference	RPA	Kavuri et al. (2022), Yuan et al. (2022)

Abbreviations: PAM (Protospacer Adjacent Motif), PFS (Protospacer Flanking Sequence), RPA (Recombinase Polymerase Amplification), LAMP (Loop-Mediated Isothermal Amplification), PCR (Polymerase Chain Reaction), RT-PCR (Reverse Transcription Polymerase Chain Reaction), HNH (Endonuclease HNH, domain), RuvC (Endonuclease RuvC domain), HEPN (Higher Eukaryotes and Prokaryotes Nucleotide-binding domain).

### 3.1 Cas9

Cas9, a prominent endonuclease, is an integral component of the type II CRISPR-Cas system, primarily employed for the precise cleavage of both strands of DNA. Its nomenclature and significance are well-documented in the scientific literature (Hsu et al., 2014), Cas9 encompasses two vital nuclease domains known as RuvC and HNH, their names derived from structural homology. A distinguishing hallmark of the Cas9 system is the PAM, situated on either side of the 3′terminal of the DNA target site. The presence of PAM governs Cas9’s search mechanism and binding specificity.

CRISPR/Cas9 has emerged as a transformative technology capable of analyzing a vast array of genes to glean essential genetic insights. Its applications extend across diverse scientific domains, including genomics, genetics, bioinformatics, and biotechnology (Yin et al., 2019). An additional pivotal facet of Cas9 is its capacity to engage specific DNA sequences via guide RNA and PAM. This unique feature has led to the development of catalytically dead Cas9 (dCas9), which lacks endonuclease activity. Researchers have harnessed dCas9 by fusing it with transcriptional activators and repressors, enabling the regulation of gene expression throughout the entire genome (Wang SW. et al., 2022). As a potent and versatile tool, CRISPR-Cas9’s genome editing technology has found utility in a multitude of fields, marking the onset of a new era in scientific research and biotechnological applications (Zhan et al., 2019).

### 3.2 Cas12

CRISPR-Cas12 has garnered significant attention in the field of nucleic acid detection due to its potential for amplified signal generation and high target recognition capabilities (Qiu et al., 2022). Notably, Cas12 proteins exhibit a remarkable ability to enhance editing efficiency, particularly in T-rich motifs, which has led scientists to explore their utility in base editing and the detection of transcriptional mutations (Hillary and Ceasar, 2023). Cas12a employs multiple checkpoints to enhance target accuracy, minimizing the risk of missing the intended target site (Paul and MontoyaCRISPR-Cas12a, 2020).

Compared to Cas9, Cas12a presents several advantages and holds promising prospects for broader applications. Cas12b, a component of the Cas12 family, possesses trans-cutting activity (Yuan et al., 2022). Cas12b falls under class II V-B endonucleases, primarily targeting double-stranded DNA (Strecker et al., 2019). A recent study (Sam et al., 2021) introduced a novel platform called TB-QUICK, which combines LAMP and CRISPR-Cas12b technology for the detection of *Mycobacterium tuberculosis*.

### 3.3 Cas13

Vi-a, also known as CRISPR-Cas13a, belongs to class II and represents a subtype within the CRISPR VI system. This RNA-guided endonuclease exhibits the ability to identify and degrade single-stranded RNA targets possessing complementary sequences. Cas13a comprises two crucial structural components: a crRNA recognition (REC) lobe and a nuclease (NUC) lobe. Cas13a possesses two interdependent ribonuclease activities, each with its own distinct catalytic site. One of these sites is responsible for the processing of pre-crRNA, while the other facilitates the cis-cleavage of target RNA and the trans-cleavage of non-specific RNA (Zhao et al., 2022).

When activated, the Cas13a protein molecule indiscriminately degrades exposed RNA molecules, encompassing both target RNA bound to the Cas13a protein and any unbound RNA in the surrounding solution. Consequently, while CRISPR-Cas13a activation is initiated by highly sequence-specific target cell binding, the ensuing Cas13a protein can cleave RNA molecules in a non-specific manner. The REC lobe of Cas13a comprises the Helical-1 domain, while the NUC lobe incorporates two HEPN and Helical-2 domains. Upon encountering the target RNA, Cas13a activation involves the movement of the HEPN1 domain towards the HEPN2 domain, subsequently leading to the binding and cleavage of target RNA sequences that are complementary during the formation of the guide-target RNA duplex (Zhang and You, 2020).

The Cas13a system has found diverse applications across numerous fields, including molecular diagnostics, gene therapy, gene editing, RNA imaging, and more. Cas13a can be heterologously expressed in mammalian and plant cells to achieve RNA knockdown of reporter genes or endogenous transcripts at levels comparable to RNA interference, albeit with improved specificity (Kordys et al., 2022). CRISPR-Cas13a, the most extensively studied system within CRISPR, has been harnessed across various domains to develop highly specific, sensitive, multifunctional, and adaptable assays. Notably, CRISPR-Cas13a serves as a potent tool for RNA knockdown and has demonstrated effects in human cancer cells (Wang Q. et al., 2019).

Cas13b, through its ability to modulate helper proteins csx27 and csx28, can divide these proteins into categories that either inhibit or enhance RNA interference activity (Sma et al., 2017). In contrast, Cas13c possesses a less pronounced function than Cas13a and Cas13b, and studies of Cas13 rarely involve the related direct homologs of Cas13c (Kavuri et al., 2022). Cas13d represents the smallest among the four Cas proteins and is one of the most potent RNA-targeting type VI systems. Type VI-D, inclusive of the Cas13d effector protein, contains accessory proteins, including a WYL domain, with one of these proteins serving as a positive regulator of target and collateral RNase activity (Yan et al., 2018).

## 4 Isothermal amplification and CRISPR-Cas fusion

### 4.1 SHERLOCK and DETECTR

In recent years, as our understanding of nucleic acid pre-amplification and CRISPR-Cas technology has deepened, researchers have developed a powerful detection platform known as SHERLOCK. This platform is capable of detecting both RNA and DNA and boasts the advantages of versatility, convenience, and high sensitivity (Kellner et al., 2019). The SHERLOCK detection process comprises three major steps: sample acquisition, amplification, and CRISPR-mediated collateral activity and detection (Zahra et al., 2023). This technology is proving to be invaluable for rapid detection applications, playing a pivotal role in human health, particularly in distinguishing between different strains of viruses and other pathogenic variants (Khan et al., 2021). SHERLOCK can rapidly identify the DNA or RNA of interest. Amplified DNA is transcribed into RNA by T7RNA polymerase, and crRNA guides Cas13a to bind to the target nucleic acid, resulting in the cleavage of reporter RNA and the release of a fluorescent signal for detection. One of SHERLOCK’s notable attributes is its rapid turnaround time. Typically, an initial RPA reaction takes 5–10 min, followed by the addition of Cas13 proteins, after which the target can be detected within just 5 min (Kellner et al., 2019).

Another diagnostic technology known as DETECTR relies primarily on the CRISPR-Cas12 lateral flow assay for the rapid detection of viral infections, offering the advantages of speed (approximately 30 min) and cost-effectiveness. Similar to SHERLOCK, DETECTR employs a system that can cleave single-stranded DNA reporter genes connected to fluorescent and quenching groups at both ends (Zhang Z. et al., 2022). DETECTR technology directs interfering proteins to release non-specific single-strand DNase activity, enabling swift detection (Chen et al., 2018).

Both SHERLOCK and DETECTR diagnostic tools share characteristics such as sensitivity, specificity, cost-effectiveness, and the absence of a requirement for complex equipment. Leveraging the versatile *in vitro* properties of CRISPR-Cas effectors, both methods convert activated nucleases into the necessary amplicons for specific nucleic acid binding events. These effectors can be linked to multiple reporter genes and combined with isothermal amplification methods for sensitive identification in various deployable formats (Mustafa and Makhawi, 2021). Earlier techniques predominantly relied on the canonical Cas9 protein from the Type II CRISPR-Cas system (Wang et al., 2016) or its modified form, the catalytically inactive or “dead” Cas9 (dCas9) protein (Brezgin et al., 2019). However, the discovery of Cas12 and Cas13 protein collateral activities has ushered in significant breakthroughs in this field (Abudayyeh et al., 2016). Cas13, in particular, exhibits target-dependent hybrid RNase activity, leading to the trans-cleavage of bystander RNA molecules, and this discovery has been harnessed for nucleic acid testing applications (Chen et al., 2018). DETECTR, in combination with isothermal pre-amplification and target sequence enrichment, not only reduces the need for stringent conditions but also enhances detection sensitivity (Mustafa and Makhawi, 2021).

**FIGURE 1:**
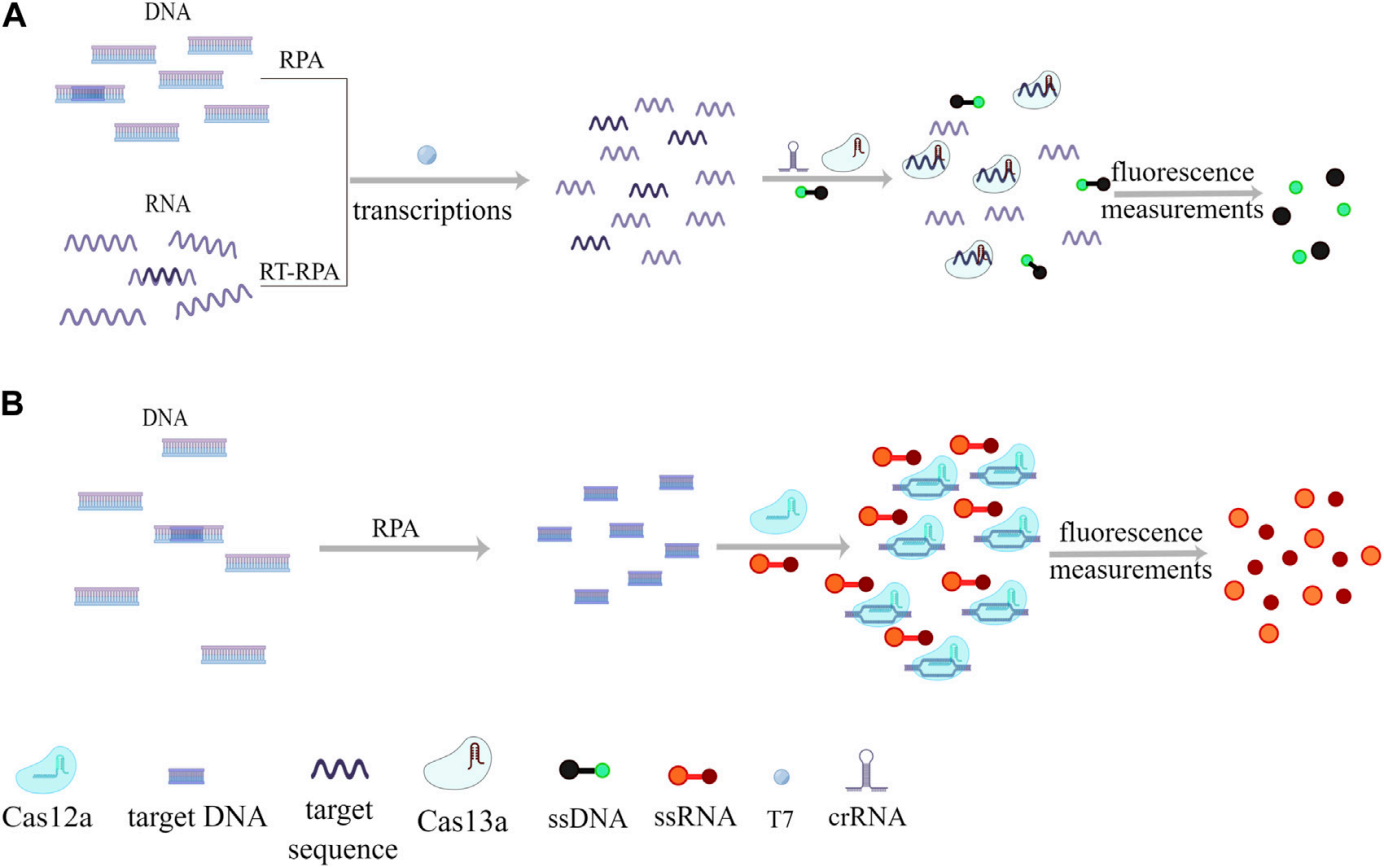
Schematic Overview of SHERLOCK and DETECTR Methods. This schematic representation contrasts the SHERLOCK and DETECTR methods, adapted from the source article (Mustafa and Makhawi, 2021). Subfigure **(A)** details the SHERLOCK method, while subfigure **(B)** illustrates the DETECTR method. Copyright for this figure is attributed to the original authors. This figure was drawn by Figdraw.

Figure 1 depicts a schematic diagram of the SHERLOCK and DETECTR methods, highlighting their shared principles with differences arising from the Cas nuclease utilized, depending on the distinct characteristics and functions of Cas13 and Cas12 (Mustafa and Makhawi, 2021).

### 4.2 One-step rapid detection: Isothermal amplification with CRISPR-Cas

The application of CRISPR in nucleic acid detection for clinical diagnosis and therapeutic purposes has been extensively documented (Pickar-Oliver and Gersbach, 2019), CRISPR technology exhibits the remarkable capability to identify target genes with high specificity, even down to a single base, enhancing the precision of nucleic acid detection. In this context, RT-RAA stands out for its efficiency in nucleic acid amplification (Wang S. et al., 2022). His efficiency not only simplifies the required instrumentation and reagents but also reduces costs, liberating detection from the constraints of specialized laboratories and trained technicians. It notably enhances detection sensitivity, making it particularly well-suited for on-site viral nucleic acid detection. However, it is worth noting that the inclusion of an additional CRISPR step may extend the assay’s reaction time, increase costs, and introduce complexity (Zhang X. et al., 2022).

Table 2 provides insights into various measures adopted by researchers to integrate isothermal amplification with the CRISPR-Cas system and the resultant effects, illustrating the ongoing efforts to refine and optimize this powerful one-step rapid detection technique.

**TABLE 2:** Enhancement of isothermal amplification combined with CRISPR-Cas for rapid pathogen detection.

Pathogens	Integrated technologies	Improvement	Fluorescent reading	Time (min)	Temperature (°C)	LOD (copy/μL)	Sensitivity (copy/μL)	References
Asfv-associatedvirus	CRISPR/Cas12a	Nested Tube Container	Clean Biosensors from CRISPR/Cas13a	30	37–39	3	3	Hu et al. (2022)
SARS-Cov-2	CRISPR/Cas13a	Nested Tube Container	Clean Biosensors from CRISPR/Cas13a	30	37–39	3	3	Hu et al. (2022)
ASFV	RAA-CRISPR/Cas12a	Both Added Simultaneously to Tube	LFA Strip with OpR Cas Platform	40	37	3.07	3.07	Xiong et al. (2022)
CaPV	RAA-CRISPR/Cas12a	Both Added Simultaneously to Tube	LFA Strip with OpR Cas Platform	40	37	1.02	1.02	Xiong et al. (2022)
SFTSV	RPA-CRISPR/Cas13a	Optimizing Single-Tank Detection Buffers	Quant-Studio1 (Applied Biosystems)	30	37	5	5	Zuo et al. (2023)
NiV	RPA-CRISPR/Cas13a	Conserved NiV Genomic Region for crRNA Design	Real-Time qPCR System	120	37	500	1000	Miao et al. (2023)

Abbreviations: Asfv (African Swine Fever Virus), SARS-CoV-2 (Severe Acute Respiratory Syndrome Coronavirus 2), ASFV (African Swine Fever Virus), CaPV (Capripoxvirus), SFTSV (Severe Fever with Thrombocytopenia Syndrome Virus), NiV (Nipah Virus), RAA (Recombinase Polymerase Amplification), RPA (Recombinase Polymerase Amplification), CRISPR/Cas (Clustered Regularly Interspaced Short Palindromic Repeats/CRISPR-associated proteins), LFA (Lateral Flow Assay), OpR (Optical Readout), LOD (Limit of Detection).

#### 4.2.1 RPA/RAA-CRISPR-Cas12a one-step application

Traditionally, most CRISPR-based nucleic acid testing strategies involve two distinct steps (Kanitchinda et al., 2020). To enable the multiple detection of foodborne pathogens, researchers have devised a microfluidic biosensor referred to as FA-MB (Finger-Actuated Microchip Biosensor). This device employs a finger-driven microchip with fluorescence detection capabilities that can be interfaced with a mobile phone (Xing et al., 2023), The key mechanism behind this technology involves conducting two separate reactions within physically isolated internal and external chambers. Subsequently, a one-way control valve facilitates the transfer of materials, reducing the risk of contamination generated during the reaction.

In a different approach, researchers have developed a rapid, highly sensitive, one-pot biosensing platform that encapsulates the CRISPR-Cas12a enzyme within a gel matrix. This setup allows for the isolation of two reagents even within the same container. In a specific experiment, this device was utilized to detect RNA from HIV, with the fluorescent signal generated serving as an indicator of the device’s effectiveness (Uno et al., 2023). Another research group has constructed a similar assay platform where the reagents and Cas12a reagents are loaded into different regions of a test tube and mixed following the completion of the RPA reaction. This process can be executed without the need to open the tube’s lid, thus minimizing the risk of contamination (Xiong et al., 2022). These innovative approaches aim to streamline and simplify the CRISPR-based nucleic acid detection process, making it more accessible, efficient, and conducive to on-site applications.

#### 4.2.2 RPA/RAA-CRISPR-Cas13a one-step application

**FIGURE 2:**
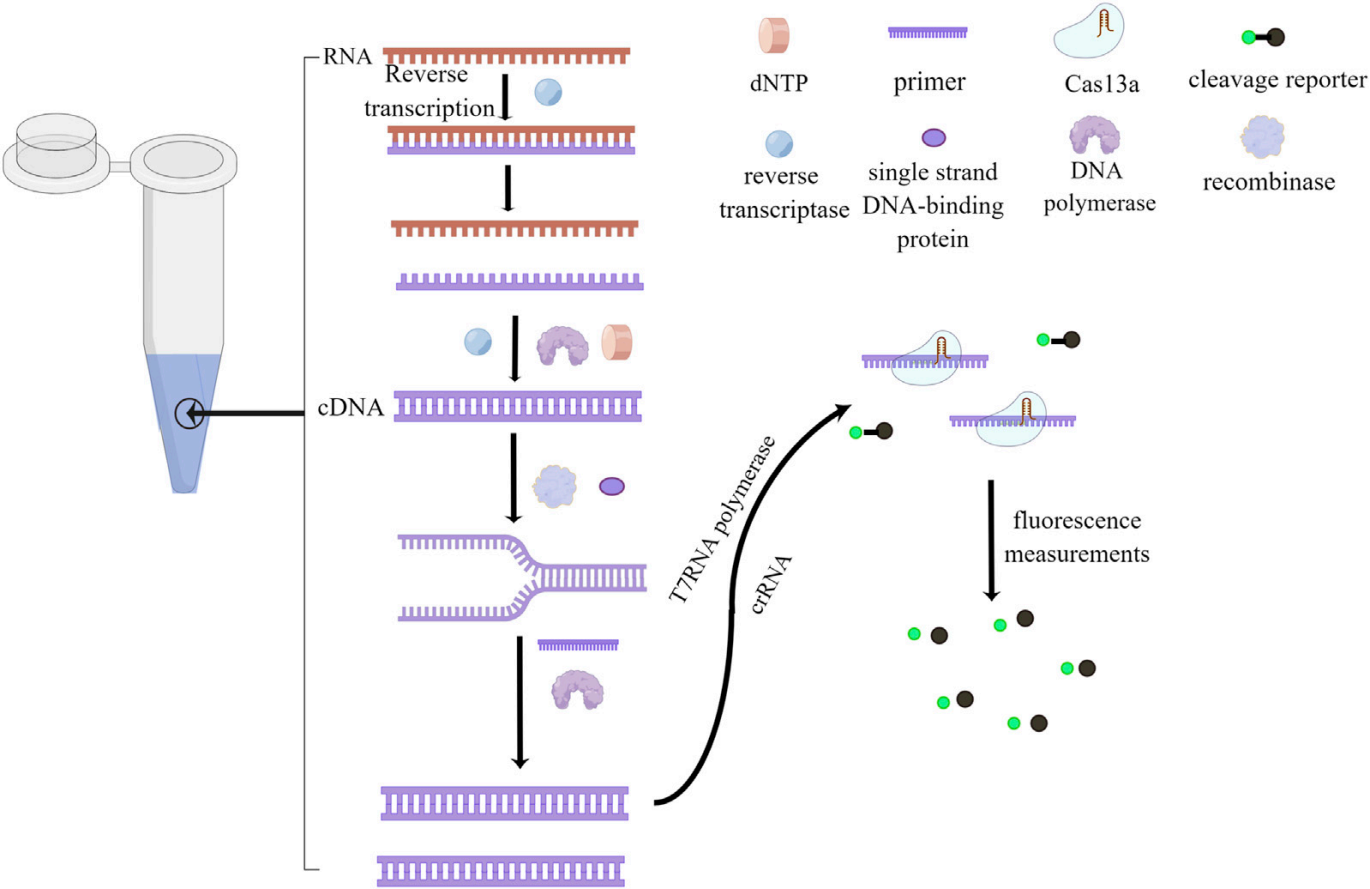
Step-by-Step Visualization of One-Tube Nucleic Acid Amplification. The process begins with the use of reverse transcriptase to convert RNA into complementary DNA (cDNA), forming a DNA-RNA hybrid chain. Reverse transcriptase then degrades the RNA, and DNA polymerase synthesizes the second cDNA strand using dNTP as a substrate. Recombinase opens the double-stranded DNA, and Single-Stranded Binding (SSB) proteins temporarily stabilize the single strand, facilitating the creation of a new complementary DNA strand through the action of primers and DNA polymerase. Subsequently, T7RNA polymerase transcribes the DNA into RNA, with crRNA guiding the Cas13a protein to bind to the target nucleic acid, resulting in the formation of a ribonuclease complex. This ribonuclease complex possesses cleavage activity, which is employed to cleave a probe. Finally, fluorescence is utilized for reading and detecting the results. This figure was drawn by Figdraw.

Figure 2 provides a clear overview of the RPA/RAA and CRISPR-Cas13a step-by-step inspection schematic, demonstrating the benefits of the technology. Efficient and highly sensitive nucleic acid detection is essential for identifying target sequences accurately. However, when employing Cas13 conjugation technology, the initial reaction speed can be slowed down due to the specific cleavage of sample nucleic acids by the Cas13 enzyme. This deceleration can impact the overall speed and effectiveness of the experiment. The primary factor contributing to this slower reaction is the involvement of T7RNA polymerase, which converts double-stranded DNA into single-stranded RNA after the completion of amplification (Arizti-Sanz et al., 2020). Furthermore, both reactions are enzymatic processes, requiring precise temperature conditions for activating the respective enzymes. Deviations from the optimal temperature range can compromise the entire reaction.

In the technical application and refinement of RPA and CRISPR-Cas13a, researchers have explored various approaches to address these challenges. One such approach involves container design, where one-pot methods for both large and small tubes have been devised to facilitate the combined use of the two techniques, thereby minimizing contamination risks. The small tube is equipped with two hydrophobic pores, allowing the transfer of contents to the large tube through centrifugation or oscillation after the completion of amplification. This design enables the experiment to proceed without the need to open the lid, reducing the possibility of contamination (Hu et al., 2022). In other studies, droplet microfluidic devices have been employed for microfluidic digital isothermal Cas13a determination, significantly shortening the overall reaction time. The core principle of this approach involves concentrating the necessary two-step processes within a closed container, employing specific isolation techniques, and ultimately achieving the desired detection outcome (Yan et al., 2014).

Another strategy involves altering the ratio and concentration of components. By meticulously designing and screening crRNA and systematically optimizing the reaction conditions, researchers have successfully developed a one-pot procedure that resolves compatibility issues between RPA and Cas13a. This approach involves integrating the RT (Reverse Transcription) step, RPA, T7 RNA polymerase transcription, and the other three steps of the CRISPR/Cas13a detection system into a single-pot method. It effectively addresses sensitivity detection challenges arising from macromolecular crowding and enzyme incompatibility within the CRISPR/Cas13a system, ensuring the stability of the quantitative reaction system (Liu FX. et al., 2022).

In the context of rapid detection of the Nepah virus using a single-tube RPA-CRISPR/Cas13a assay (Miao et al., 2023), specific components were utilized, including 13 nM LwaCas40a, 13 nM crRNA, 20 nM quenched-fluorescence RNA reporter genes, and 125 U RNASE inhibitor. A ratio of 10 mM rNTP, 2 μL cDNA template, and 1 μL B buffer was injected into the sample to activate these reactions, yielding positive results. Additionally, in the detection of SFTSV, the optimized single-tube detection buffer significantly enhanced the sensitivity, specificity, and Limit of Detection (LOD) of the single-tube reaction (Zuo et al., 2023). These innovative strategies demonstrate ongoing efforts to streamline and enhance the efficiency of one-step nucleic acid detection methods, making them more practical and accessible for various applications.

## 5 Application in pathogen detection

In recent years, the integration of RPA/RAA and CRISPR-Cas technology has made significant strides in pathogen detection. For instance, SHERLOCK technology has demonstrated its ability to differentiate between specific strains of Zika and Dengue viruses, enabling the distinction of causative strains (Gootenberg et al., 2017). Researchers have applied SHERLOCK, employing both two-step and one-step reactions, to detect parasite RNA and diagnose Human African Trypanosomiasis (HAT) (Sima et al., 2022). The DETECTR technique, in the detection of human papillomavirus (HPV), has successfully differentiated types 16 and 18 with high accuracy and in a short detection time (Chen et al., 2018).

**FIGURE 3:**
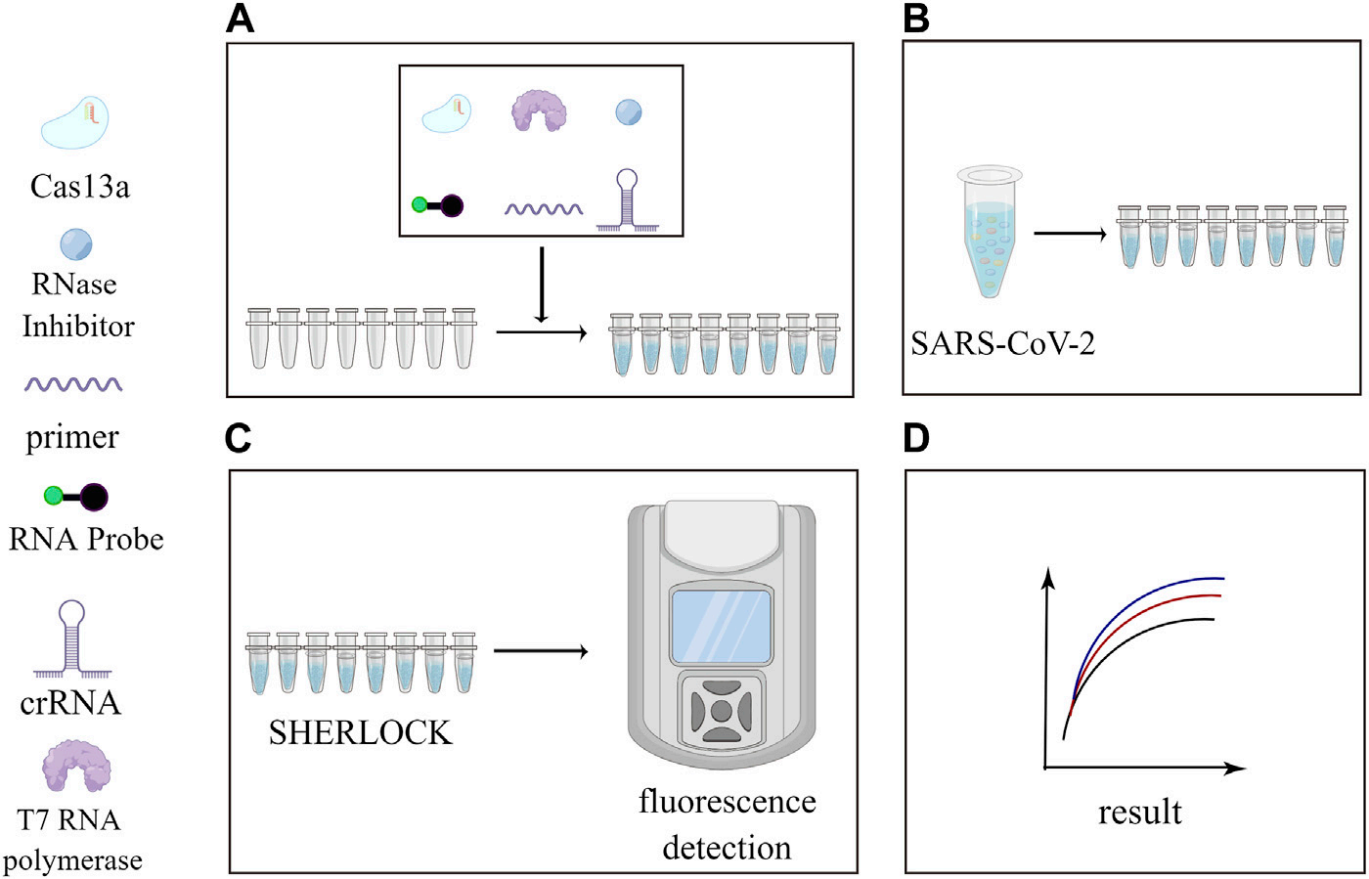
Application of SHERLOCK in the detection of SARS-CoV-2. This figure was drawn by Figdraw. Subfigure **(A)**: Cas13a, cleavage reporter, RNase Inhibitor, crRNA, primer, T7 RNA polymerase and other reagents were added to each of the eight consecutive tubes at a certain ratio. Subfigure **(B)**: Add a quantity of SARS-CoV-2 to each tube. Subfigure **(C)**: Perform the reaction using the SHERLOCK platform and view the fluorescence curve in the fluorometer. Subfigure **(D)**: Fluorescence curves obtained.

With the continuous refinement and maturation of SHERLOCK and DETECTR technologies, they have also played a pivotal role in the detection of the Coronavirus Disease 2019 (COVID-19) in the recent past (Zhou et al., 2022). SHERLOCK technology, known for its robustness, has been effectively utilized to recognize SARS-CoV-2 RNA, offering improved target specificity (Khan et al., 2021). Some researchers have employed the CRISPR-Cas12-based DETECTR assay for the detection of SARS-CoV-2 (Broughton et al., 2020), The development of DETECTR’s single-tube assay platform has enabled the rapid detection of SARS-CoV-2 in approximately 50 min (Sun et al., 2021). The combination of CRISPR technology with isothermal nucleic acid amplification, followed by visualization using lateral flow immunoassays (LFIA), has also found applications in SARS-CoV-2 detection (He et al., 2022). Taking Figure 3 as an example, we summarize the clinical applications of the SHERLOCK technology during the COVID-19 and found that the SHERLOCK technology made a great contribution to the detection of outbreaks.

However, it is important to note that these technologies still face certain challenges, including multistep nucleic acid amplification and commercialization limitations (Zahra et al., 2023). Nevertheless, the combined application of RPA/RAA technology and CRISPR-Cas has significantly altered the landscape by addressing environmental and site constraints, reducing costs, and enhancing sensitivity.

In summary, this section has highlighted some applications of one-step rapid detection technology based on isothermal amplification combined with the CRISPR-Cas system. These innovations have effectively addressed issues such as complex procedures, aerosol contamination, and low sensitivity in CRISPR nucleic acid detection. While these technologies may not be fully matured and have limited applications at present, they have shown promising sensitivity in detecting various pathogens, including ASFV and CAPV-associated viruses, NiV, SFTSV, and HPV16. These advancements have provided crucial support for clinical diagnosis and treatment in the field of pathogen detection.

## 6 Discussion

In the realm of isothermal amplification, LAMP, RPA, and RAA stand out as the most prevalent techniques, each possessing distinct advantages and drawbacks. LAMP, renowned for its high specificity and resistance to interference, boasts a stable reaction system that remains unaffected by extraneous and interfering fragments within the sample (Soroka et al., 2021). Its straightforward visual identification and naked-eye observation capabilities are additional merits. Nonetheless, LAMP faces limitations in amplifying long DNA strands and demands precision. While its instrumentation costs are relatively lower, reagent expenses can be a concern.

RPA technology offers rapid detection, often without the need for skilled personnel. It allows for multiple amplification reactions in a single tube and employs a simple primer design, thereby ensuring high sensitivity and specificity. Unlike LAMP, RPA operates without the necessity of heating equipment like water baths. However, RPA is not without its shortcomings, requiring product purification before imaging and posing challenges in avoiding nonspecific amplification during experiments. RAA, sharing similarities with RPA in principle, exhibits comparable strengths and weaknesses.

Common Cas nucleases include Cas9, Cas12, and Cas13, each possessing distinct characteristics. The CRISPR-Cas13a system excels in recognizing and cleaving RNA, with applicability to various RNA viruses. Cas13a finds extensive utility in molecular diagnostics, gene therapy, gene editing, and RNA imaging. Cas9 and Cas12a, as multi-domain effector proteins with double-leaf structures, offer broad applicability but are reliant on the host cell’s DNA repair mechanism (Paul and MontoyaCRISPR-Cas12a, 2020). In contrast, Cas12b, although smaller in size, demonstrates high specificity, making it invaluable in the genetic engineering (Yang et al., 2023). Cas13 proteins excel in sequence-specific cleavage of single-stranded RNA molecules, enabling precise regulation of RNA products and mitigating unintended pleiotropic effects (Kavuri et al., 2022).

Comparing various amplification methods, the combination of RPA/RAA with CRISPR-Cas technology exhibits clear advantages over other combinations. PCR combined with CRISPR-Cas proves less suitable due to the complexity of the PCR process, intricate primer design, and the requirement for a temperature range (approximately 60°C–65°C) divergent from the CRISPR-Cas system’s needs (Li et al., 2022). Isothermal amplification technology, on the other hand, simplifies equipment requirements and enhances sensitivity and specificity when coupled with CRISPR-Cas technology. While LAMP offers robust amplification capabilities with low nucleic acid concentration detection limits (Atceken et al., 2022). It faces constraints regarding DNA target sequence length and primer design complexity. In contrast, the merits of RPA/RAA become more pronounced. Operating at 37°C without necessitating specialized equipment and featuring rapid amplification, RPA/RAA proves a more suitable choice.

The refinement and application of RPA/RAA techniques combined with CRISPR-Cas technology are ongoing. SHERLOCK, noted for its ease of use and high sensitivity, can detect both RNA and DNA. DETECTR, a CRISPR-Cas12-based lateral flow detection method, proves valuable in identifying viral infections. Researchers have made substantial strides in unifying these technologies, from integrating pre-amplification with CRISPR-Cas in SHERLOCK to harnessing Cas12a′s non-specific single-stranded DNA degradation ability for DETECTR development. Most improvements center around optimizing containers, physically isolating components, or leveraging external environmental changes for isolation. Researchers have also fine-tuned test formulations by modifying or adjusting the concentration and dosage of specific components. The combination of RPA’s robust amplification and CRISPR’s precision holds significant promise, reducing costs, enhancing user-friendliness and stability, and importantly, minimizing contamination associated with transferring between tubes. Fine-tuning operational steps and materials further enhances rapid detection technology. Table 2 demonstrates that the microfluidic digital isothermal Cas13a assay utilizing a droplet microfluidic device offers the highest sensitivity. The improved fluidity in droplet microfluidic devices enhances reagent binding and reduces reaction times. Additionally, this technology can be combined with commercial equipment, expanding its accessibility to non-professional users and broadening its application scope (Liu FX. et al., 2022). Other improvements in physical isolation and formulation optimization have yielded positive results. Hence, we anticipate significant opportunities and possibilities for further advancements in combined technologies in the future.

## 7 Conclusions and future prospects

Pathogens, the microscopic invaders responsible for infections and diseases in the human body, have prompted ongoing advancements in detection technologies. This paper has provided an overview of the RPA/RAA and CRISPR-Cas technologies, with a focus on their combined application in pathogen detection—a crucial reference for researchers in this field. Methods that integrate amplification with nuclease activity to offer additional detection and amplification signals have opened new avenues for pathogen detection (Wang et al., 2023).

RPA/RAA technology represents a relatively recent approach to isothermal amplification, offering distinct advantages over traditional PCR techniques in terms of cost, portability, and specificity. The high specificity and reliability of RNA nucleic acid detection via Cas proteins are noteworthy. When coupled with RPA pre-amplification, this technology not only eliminates unnecessary non-specific amplification signals but also enhances the sensitivity (Chen et al., 2020). This combined technology has emerged as a potent detection technique, finding widespread application in detecting various viruses, promising substantial market potential. Compared to alternative methods, this approach operates at room temperature, requires minimal expertise, offers rapid results, and demonstrates high sensitivity and specificity.

To address issues related to contamination, low efficiency, and complex procedures in the detection process, researchers have devised various measures for improvement. Many have achieved single-tube, one-step detection through container enhancements and alterations to the external environment.

The aftermath of the COVID-19 pandemic has left an indelible mark on the field of pathogen detection, catalyzing a renewed and intensified focus on the development of cutting-edge diagnostic techniques. In response to the challenges posed by the pandemic, researchers worldwide have embarked on a mission to create innovative technologies that meet several critical criteria: user-friendliness, cost-effectiveness, and the ability to deliver rapid and accurate results.

In this pursuit, the ongoing evolution of the RPA/RAA and CRISPR-Cas combination technology has emerged as a beacon of hope and progress in the realm of pathogen detection. While this technology is still in its early stages of development, its potential is nothing short of transformative. As researchers delve deeper into the intricacies of these two powerful methodologies, they are continually fine-tuning and optimizing various aspects of their systems, from fundamental conditions to specific parameters.

The ultimate goal of this relentless innovation is to achieve one-step pathogen detection-a seamless and efficient process that can be executed with ease by professionals and non-professionals alike. Such a breakthrough would represent a significant milestone in the field, streamlining the detection of pathogens in diverse settings, from clinical laboratories to remote field locations. By eliminating complex steps and minimizing the risk of contamination, this innovation has the potential to revolutionize the way we identify and respond to infectious diseases.

As the RPA/RAA and CRISPR-Cas combination technology matures and refine its capabilities, it is poised to assume a pivotal role in the future of pathogen detection. With its ability to deliver rapid, specific, and sensitive results at room temperature and without the need for extensive equipment, this technology holds the promise of becoming a versatile and indispensable tool in the ongoing battle against infectious diseases. The collaborative efforts of scientists and researchers worldwide continue to push the boundaries of what is possible in pathogen detection, offering hope for a safer and more resilient future (Li et al., 2023).
